# Cementless Total Hip Replacement for the Management of Severe Developmental Dysplasia of the Hip in the Middle Eastern Population: A Prospective Analysis

**DOI:** 10.3389/fsurg.2016.00031

**Published:** 2016-05-30

**Authors:** Mohamed A. Imam, Ismail Fathalla, James Holton, Mohamed Nabil, Fadhil Kashif

**Affiliations:** ^1^Department of Orthopaedic, Faculty of Medicine, Suez Canal University, Ismailia, Egypt; ^2^South West London Elective Orthopaedic Centre, Epsom, UK; ^3^Royal Orthopaedic Hospital, Birmingham, UK

**Keywords:** dysplasia, hip, Crowe, surgery, DDH

## Abstract

**Introduction:**

In the Middle East, severe developmental dysplasia of the hip with subsequent high dislocation is often seen. We assessed the efficiency of total hip replacement (THR) with subtrochanteric shortening femoral osteotomy and trochanteric advancement in this population.

**Methods:**

This prospective study assessed 25 female patients with symptomatic and severe (Crowe IV). Pre- and postoperative Harris hip score (HHS) and Oxford hip score (OHS) were performed alongside assessment of leg length discrepancy (LLD) and the ability to sit in a cross-legged position.

**Results:**

The mean HHS and OHS improved pre-operatively at 1 and 10 years, respectively (*p*-value < 0.001). The mean postoperative LLD was 3 mm (0–8 mm). Functionally, 22/25 patients were able to sit cross-legged. None of the 25 hips underwent revision during this period.

**Conclusion:**

Total hip replacement with subtrochanteric shortening osteotomy in combination with trochanteric advancement is sufficient for the management of Crowe type IV hips in this population.

## Introduction

Severe and untreated developmental dysplasia of the hip (DDH) with subsequent high dislocation is not frequently encountered in the developed world due to hip screening and early active treatment. However, in developing countries, the end stage of DDH sequelae is more frequently encountered and ultimately requires total hip replacement (THR) as hip preservation procedures are not feasible. These patients are often young and the procedure is technically challenging (1–3). Many studies have highlighted the difficulty in regard to preoperative planning, preparing of the true acetabulum, restitution of the offset, and the correction of leg length discrepancy (LLD) (2, 4). Subtrochanteric femoral shortening osteotomy has been proposed as a concomitant procedure that is carried out simultaneously to overcome this problem (2–7). This also helps avoid stretching of the neurovascular structures once the femoral head is close to the anatomical position.

Severe DDH (Crowe Type IV) (1) are usually associated with anatomical abnormalities. Commonly, those patients present with a shallow acetabulum with proximal dislocation of the hip with increased anteversion of the femoral neck together with poor bone quality (8). Accordingly, there is increased risk of postoperative complications when compared to osteoarthritic hips. There is an increased risk of sciatic nerve palsy (9–11), infection (10, 12, 13), dislocation (2, 14), intraoperative fracture of the femur (15), and consequently the need for revision surgery (16, 17).

There is paucity in the literature correlating the survivorship of the prosthesis, functional outcomes, and the component orientation in primary THR undertaken for severe DDH hips. Morag et al. compared the functional outcome and survivorship of the prosthesis in relation to the positioning of the acetabulum (16). This study found that the height of the cup was found to have a statistically significant correlation with functional outcome. The greater cup height predicted a worse functional outcome but also risk of revision for loosening. To achieve the best outcome, the study recommended placing the cup as close the anatomical position as possible (16). Cementless THR with subtrochanteric femoral shortening osteotomy has been proposed for management of these cases. Tokgozoglu and Caglar used this technique with distal advancement of the greater trochanter to improve the abductor mechanism and avoid non-union in 91 hips (7). At 8 years, patients had improved Merle d’Aubigne scores; however, this was balanced against a 10% risk of failure (7). Yalcin et al. assessed cementless THR with femoral shortening osteotomy in 44 hips with an average age of 43.2 years (18). The Harris hip score (HHS) improved 45 points at an average of 62 months follow-up. Again, 23% experienced complications, including five non-unions, two dislocation, two superficial infections, and one acetabular displacement. No patients experienced neurovascular injury (18). A review of THR in adult DDH by Sanchez-Sotelo highlighted that THR relieves pain and improves function although the mid-long-term outcomes are not as good due to the technical difficulties posed by these complex patients (19).

This is the first study reported for this cohort of patients in a Middle Eastern population. The Middle East is a distinct and dissimilar region where politics and religion significantly affects daily life, including health care. There are unique racial, cultural, and socioeconomic factors that potentially affect outcome and require special attention when managing these hips. There is the need to undertake more demanding activities and postures in this population. Sitting cross-legged or sitting on the floor are routine living activities that would constitute a common expectation in patients having their hips replaced in the Middle East (20). Additionally, the reported THR techniques reported in western populations do not account for the persistent cross-legged sitting position. Therefore, there is a unique void in the literature on THR in severe DDH in Eastern population to meet the lifestyle requirements of Middle Eastern patients.

We have prospectively assessed the efficiency of the combination of cementless THR and subtrochanteric shortening femoral osteotomy with trochanteric advancement in patients with Crowe type IV DDH in a Middle Eastern Population.

## Materials and Methods

This study protocol was approved by the local institutional review board. This prospective study was conducted in three hip reconstruction units between January 2005 and October 2012. After fulfilling our inclusion/exclusion criteria, 25 consecutive hips in 25 patients were recruited for this study. Two patients were excluded from final analysis as they were lost to follow-up and two patients asked to withdraw from the study. This left 21 patients (all females) who were available for the final analysis. The STROBE guidelines were used to ensure the reporting of this observational study (21). All fulfilled our selection criteria of symptomatic dislocated degenerative hips with history of severe DDH (Crowe type IV), with no absolute medical contraindications to surgery, able and willing to provide written consent and were compliant to radiographic and clinical follow-up post surgery. Patients with neuromuscular compromise in the symptomatic extremity were excluded. Ethical approval was obtained prior to the initiation of the study. All patients were given information sheets regarding the proposed procedure and the study protocol.

### Pre and Postoperative Assessment

**Figure 1:**
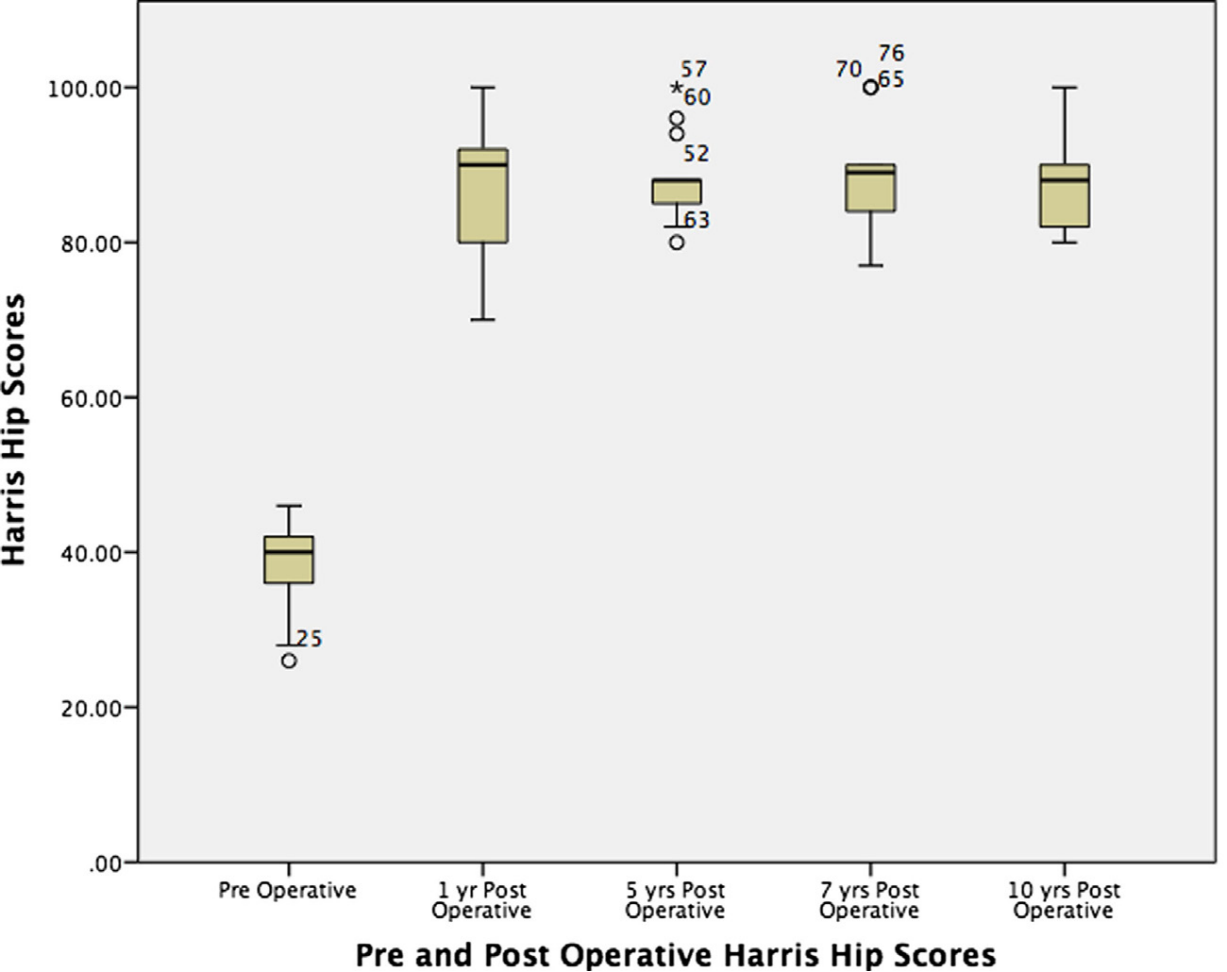
**Boxplot and whisker diagram in the change of Harris hip score pre- and postoperatively over time**.

**Figure 2:**
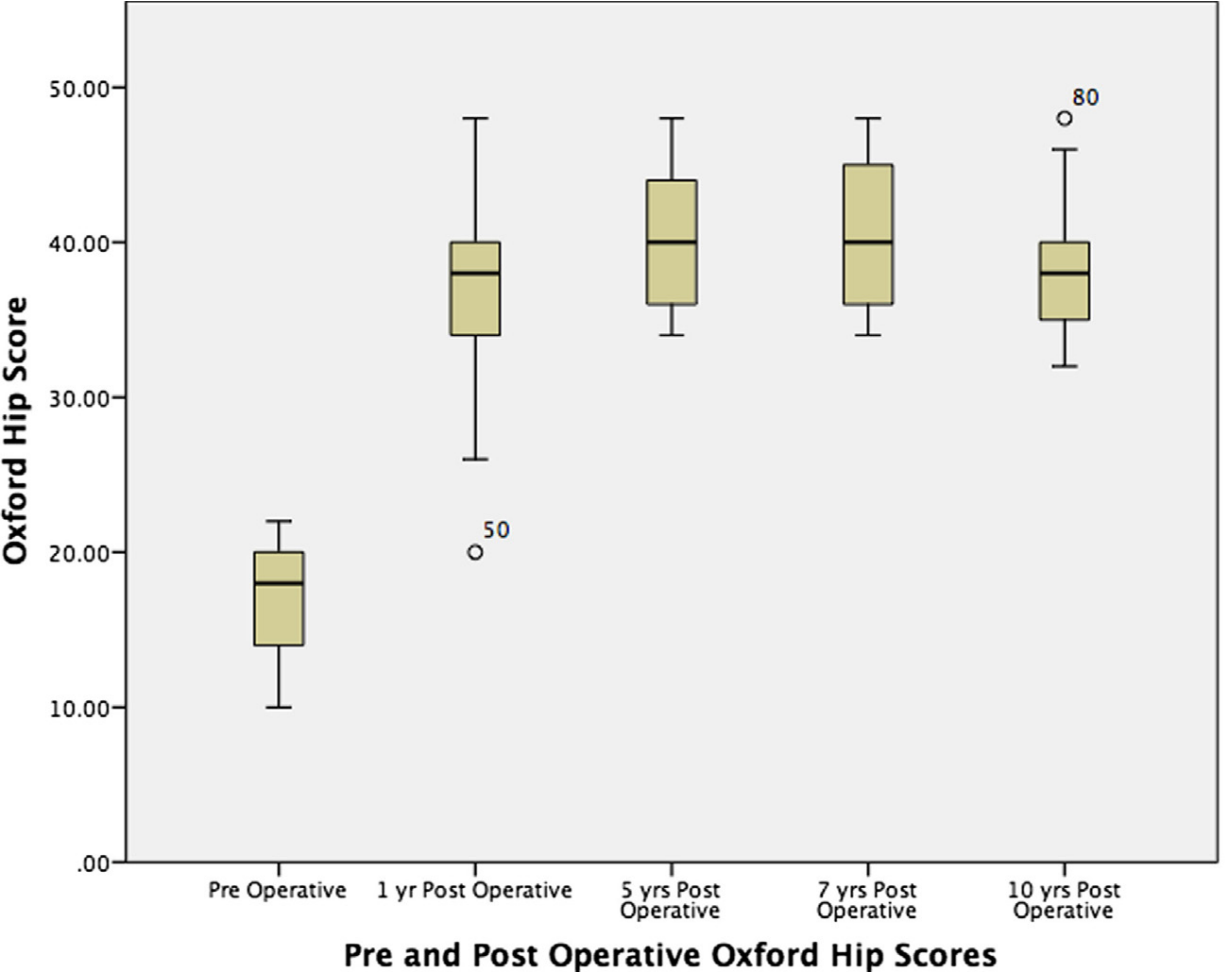
**Boxplot and whisker diagram in the change of Oxford hip score pre- and postoperatively over time**.

The patients’ demographic data: age, sex, gender, BMI, preoperative LLD, and Crowe grades of both hips were recorded (Table 1). All patients were evaluated before and after surgery with HHS and Oxford hip scores (OHS). Additionally, the analysis examined the change in HHS and OHS over the course of follow-up (Figures 1 and 2). The LLD was clinically evaluated measuring the distance between the anterosuperior iliac spine and the medial malleolus on both sides.

**Table 1:** **Summary of patients’ demographics and preoperative findings**.

Patient	Age	BMI	Side	Gender	Symptomatic hip	Contralateral hip	Leg length discrepancy (mm)
1	24.3	22	Right	Female	Crowe IV	Crowe I	38
2	29.4	25	Left	Female	Crowe IV	Crowe II	40
3	27.1	28	Left	Female	Crowe IV	Normal	30
4	22.5	32	Left	Female	Crowe IV	Crowe I	42
5	33.4	35	Left	Female	Crowe IV	Crowe I	46
6	31.3	29	Left	Female	Crowe IV	Normal	38
7	30.3	27	Left	Female	Crowe IV	Crowe III	38
8	31.2	28	Right	Female	Crowe IV	Normal	40
9	32	38	Left	Female	Crowe IV	Crowe III	42
10	33	35	Right	Female	Crowe IV	Crowe III	36
11	31.3	34	Right	Female	Crowe IV	Normal	40
12	29.4	38	Left	Female	Crowe IV	Normal	40
13	31.1	25	Left	Female	Crowe IV	Normal	46
14	26	22	Left	Female	Crowe IV	Crowe III	50
15	29.3	35	Left	Female	Crowe IV	Crowe III	48
16	38.2	38	Left	Female	Crowe IV	Normal	36
17	24.1	35	Left	Female	Crowe IV	Crowe II	38
18	29.6	36	Left	Female	Crowe IV	Normal	38
19	39	28	Left	Female	Crowe IV	Normal	36
20	31.1	35	Left	Female	Crowe IV	Crowe III	36
21	30.2	33	Left	Female	Crowe IV	Crowe III	42

A CT scan with 3D reconstruction was performed to confirm the true dimension of the native acetabulum and to assess for proximal femoral deformities. Transverse femoral subtrochanteric shortening osteotomy was done in all cases to accomplish stable reduction and avoid traction injury to the sciatic nerve. Radiological evaluation of union of femoral shortening osteotomy was done at 3 and 6 months postoperatively.

### Operative Technique

All patients had Crowe type IV dysplasia and underwent cementless THR (Korus cementless HA-coated stem, Fin II HA-coated cup, Bio-Impianti, Milano, Italy) accompanied with femoral shortening osteotomy in the same setting. We utilized metal heads (size 28 mm) on highly cross-linked polyethylene liners in all cases. Patients were positioned in the lateral position and a modified Hardinge approach to the hip was performed. After incision of the skin, subcutaneous fat and the fascia lata, an anterolateral flap osteotomy of the greater trochanter was undertaken using an oscillating saw and completed with an osteotome to keep the hip abductors intact and maintain function. Distal advancement of the greater trochanter was undertaken in all cases. Identification and preparation of the true anatomical acetabulum was performed. A cementless cup with 2–3 screws with a target inclination angle of ~35° was used.

The femoral osteotomy was performed 1 cm distal to the lesser trochanter. The proximal femur was prepared, and the stem implanted, acting as a stabilizer of the osteotomy. A trial of reduction was then performed to assess whether further femoral shortening was required. The femoral head was assembled to the stem and reduction achieved. The subtrochanteric osteotomy was secured with a tension band, and the trochanteric flap was re-attached using screws and wire sutures. Anatomical closure in layers over a vacuum suction drain was performed. At the end of the surgery, the patient was turned into a supine position and the contracture of the adductor tendons was assessed as well as the motion in abduction, in order to evaluate the necessity of percutaneous partial adductor tenotomy.

### Postoperative Care

A standard rehabilitation program initiated immediately postoperatively but full weight bearing was avoided for 8–12 weeks until there were radiographic signs of union at the osteotomy site. From this point, abductor strengthening exercises were commenced. Antibiotic and thrombo-embolic prophylaxis was given as per local institutional protocol.

### Statistical Methods

Statistical analysis was performed using SPSS software (Version 14; SPSS Inc., Chicago, IL, USA). Data were checked for normality of distribution using the Kolmogorov–Smirnov test. All data were non-parametric and, therefore, the Mann–Whitney *U* test was applied with predetermined *p*-values <0.05 considered statistically significant.

## Results

The mean HHS improved from 38.4 points preoperatively to 88 and 88.6 points at 1 and 10 years postoperatively, respectively (*p*-value < 0.001). The mean OHS improved significantly from 17 points pre-operatively to 37.1 at 1 year, and 38.6 points at 10 year (*p*-value <0.001). After the first year, there was no significant change in values over time.

All patients were females with a mean age of 30.5 (22–39) years with an average BMI of 30 (22.9–39) at the time of surgery. Seven patients had Crowe type III dysplasia in the contralateral hip (Table 1). The LLD before surgery ranged from 30 to 50 mm with a mean of 40.2 mm. The intraoperative femoral resection ranged from 20 to 27 mm with a mean of 23.5 mm. Adductor tenotomy was performed in 15/21 (71%) patients. The postoperative LLD ranged from 0 to 8 mm with a mean of 3 mm. The duration for radiological signs of union at the osteotomy site to appear ranged from 3 to 5 months with a mean of 3.79 months. 22/25 (88%) patients reported that they were able to either sit cross-legged or sit on the floor.

### Complications

Five patients (five hips) had radiological evidence of heterotopic ossification without any clinical symptoms in the first 6 months of radiological follow-up. Two patients had dislocation after 3 months post surgery, which was managed successfully with closed reduction under anesthesia. Three patients were treated successfully for a possible deep vein thrombosis and one was treated for pulmonary embolism after surgery. None of the patients reported persistent nerve injury at 6 months post surgery. Three patients had superficial wound infection postoperatively that responded well to the appropriate antibiotics according to culture and sensitivity. None of the 21 hips were revised.

## Discussion

The Middle East is a heterogeneous region where late presentation of DDH is common, especially in females. Family structure and deprivation have increased the number of neglected cases more commonly in woman. It is important that researchers in healthcare have an understanding of different socioeconomic, cultural, and religious factors involved in most aspects of life. The high prevalence of DDH has been linked to consanguinity in the Middle East where 25–49% of DDH cases are from consanguineous parents (22). The common practice of swaddling children may also impact hip development in this geographic area (23). Moreover, the neonatal screening of DDH in this region is modest, together with the lack of awareness and the scarcity of routine neonatal hip examination has led to increased incidence of delayed diagnosis especially in more deprived areas.

Few studies have been carried out on the kinematics of the hip to undertake the sitting cross-legged position. Kapoor et al. (24) and Hemmerich et al. (25) reported that the range of motion required to perform the cross-legged posture were greater than that delivered by the available THR prostheses. This observation was also proved by rehabilitation studies, indicating patients struggle to achieve this after THR (26). The cross-legged sitting position demands a greater range of motion with hip flexion, abduction, and external rotation (27). Patients in our cohort were satisfied with their functional outcomes with 88% (22/25) of patients being able to either sit cross-legged or sit on the floor.

Total hip replacement for high dislocation of the hip is not a commonly performed procedure even in specialized hip reconstruction centers. Historically, Charnley and Feagin recommended the avoidance of THR in patients with severe DDH because of inadequate bone stock (28). However, the use of cementless THR simultaneously with shortening femoral osteotomy for management of advanced osteoarthritis secondary to severe DDH has been reported (2–4, 6).

A key consideration in Crowe IV hips is where to place the center of rotation of the new hip replacement. Most authors agree that the true acetabulum is the favorable choice biomechanically (2, 4, 29, 30). Some authors suggested that positioning the acetabular component in a higher position is associated with early loosening, mainly due to poor bone stock that is incapable of providing the adequate support needed (31). Increased rate of loosening was also observed when utilizing cemented acetabular components when placed above the true acetabulum (32, 33).

Trochanteric osteotomy was performed in all cases, which provided good access to the acetabulum and with advancement aimed to improve the abductor function.

The high riding femur is a major problem in patients with type IV Crowe hips as it complicates the reduction after restructuring the acetabulum in the native position. We believe femoral shortening with a subtrochanteric osteotomy is essential in those patients to achieve stable reduction and protect against neurovascular traction injury. Additionally, it reduced the mean LLD post surgery. The transverse subtrochanteric osteotomy is the simplest to perform, while oblique or chevron osteotomies provide more rotational stability (7).

We have not reported any nerve palsy in our series similar to other reports (2, 4, 7). Many authors report increased risk of sciatic nerve palsy post surgery from 3 to 15% in dysplastic hips compared to 0.5 to 1% in degenerative hips (9–11). Also higher risks of infection (10, 12, 13) and dislocation (2, 14) have been reported. The increased risk of revision is a major concern (16, 17). Despite the fact that we do not report any revisions in our series, revision THR with a previous diagnosis of DDH is a challenging procedure. Higher hip center positioning and deficient acetabular bone stock in the primary THR renders the revision more technically demanding and usually associated with poorer outcomes (16, 17).

A limitation of this study is the relatively short follow-up period in some of the cases. However, we plan to continue to follow-up this cohort of patients. Another limitation is the relatively small number of hips included in this study, although severe DDH is relatively common in the Middle East (34). Moreover, the patients lost to follow-up could be a source of bias in this report (35).

We report 100% survivorship using uncemented THR for management of severe DDH in the Middle Eastern Population at 2–10 years follow-up. This is the first report from this region. This study confirms the reliability of using simultaneous femoral shortening osteotomy in these cases will significantly reduce the risk of neurovascular injuries. A precise preoperative assessment is crucial to achieve good results in this type of procedure.

## Author Contributions

MI: initial design and lead on the study, and involved in data collection and analysis. MN: involved in the initial design and data collection with proof reading and drafting. JH: drafting and revising of the manuscript ready for submission, and submitting and corresponding author. IF: involved in the initial design, drafting, and revising alongside proof reading of the manuscript. FK: involved in the study design, proof reading of the manuscript draft, and senior author.

## Conflict of Interest Statement

The authors declare that the research was conducted in the absence of any commercial or financial relationships that could be construed as a potential conflict of interest.
